# Editorial: Clinical guidelines in OCD: applications and evaluation

**DOI:** 10.3389/fpsyt.2024.1373923

**Published:** 2024-02-07

**Authors:** Andrea Poli, Daniel Lucas da Conceição Costa, Marco A. Grados

**Affiliations:** ^1^ Department of Clinical and Experimental Medicine, University of Pisa, Pisa, Italy; ^2^ Institute of Psychiatry, Hospital das Clinicas, Faculdade de Medicina, Universidade de Sao Paulo (HCFMUSP), Sao Paulo, Brazil; ^3^ Deparment of Psychiatry & Behavioral Sciences, Division of Child & Adolescent Psychiatry, Johns Hopkins University School of Medicine, Baltimore, MD, United States

**Keywords:** OCD (obsessive-compulsive disorder), event related potentials - ERP, executive functions, OCD insight, self-schemas

Obsessive−compulsive disorder (OCD) represents a highly heterogeneous diagnostic entity that still poses clinical assessment and treatment challenges (1–5). Currently, OCD is believed to represent a spectrum of potentially overlapping symptom dimensions, rather than mutually exclusive symptom categories. OCD symptom dimensions have been shown to be continuous with typical OC phenomena and manifest in OCD patients as well as in the general population (6–8). OCD is often undiagnosed in primary care settings and frequently undertreated; hence, screening, diagnosis, and treatment options are continuously updated (9, 10), even for the perinatal period (11). In addition, neurobiological research approaches show that cortico-striatal-thalamic-cortico circuits, in particular orbitofrontal cortex and striatum regions (caudate nucleus and putamen), are responsible for mediating OCD symptoms. Interestingly, these brain regions are targeted by cognitive-behavioral therapy and appear to restructure, modify, and transform in response to the intervention with symptom improvement, supporting a neurobiological component of OCD (4, 12, 13). Hence, an understanding of the neurobiological machinery implicated in OCD can be informative for the assessment, diagnosis, evaluation, or treatment of OCD.

This Research Topic collects 2 articles which develop our understanding of how OCD is able to affect cognitive processes such as attention, insight skills and quality of life. In addition, 2 articles aim to clarify, using an event-related potential paradigm, the emotional components involved in contamination-related OCD, and the role of negative self-schemas in driving pathological doubt in OCD.

Doolub et al. investigate the links between treatment-resistance, executive function and working memory, and the severity of OCD symptoms in 66 patients with OCD. In individuals with OCD, treatment resistance is associated with lower inhibition performance on the Stroop test, as the sole predictor of treatment resistance. Since the Stroop test mainly taps the inhibition of prepotent responses this suggests that high treatment-resistance is associated with patients’ inability to block automatic cognitive processes and that treatment-resistant patients are less prone to prevent compulsions than their non-resistant counterparts. Hence, the Stroop test, or other tests measuring inhibition, might be helpful in clinical settings to predict OCD clinical outcomes. This study confirms, in addition, that patients with OCD display broad, but relatively moderate alterations of executive and working memory abilities compared to other psychiatric pathologies, and that these cognitive deficits are not linked to the severity of the illness.

Wolf et al. explore the course of insight and its association with OCD severity and quality of life among 253 individuals with OCD participating in a four-year prospective naturalistic study, the Netherlands Obsessive Compulsive Disorder Association (NOCDA) Study. Of participants with available insight data, changes occurred in 70% of cases and insight was most variable in participants with poor insight. In addition, the authors found that changes in insight were correlated with changes in OCD severity with a small to medium effect size, indicating that improvement in insight and OCD severity are associated. However, changes in insight in the first two years were notsignificantly predictive of OCD severity or quality of life after four years.

A convenience sample of 45 participants was investigated by Golkhatmi et al. in order to examine the emotional components of event-related potentials (ERPs) in individuals with contamination OCD and compare them with a healthy control group. The sample consisted of 30 individuals diagnosed with contamination-type OCD and 15 individuals in a healthy control group. Results highlighted that patients with the contamination subtype OCD (C-OCD) may show a sensitivity of N2 and P3 components of ERPs to emotional and cognitive impairments of C-OCD. The authors highlight the potential of N2 and P3 components of ERPs as markers and useful tools in therapeutic interventions.

Schoeller discusses how recent evidence may suggest a model of OCD as stemming from flawed high-level priors contained in a dysfunctional self-model, and how compulsions are conceptualized as artificially generated evidence to resolve higher order self-uncertainty. This process may be captured by the mechanics of active inference, or the idea that the brain continuously generates and updates a model of the world using probabilistic, predictive processing. This model leads to the prediction that compulsive rituals should continue as long as self-uncertainty and flawed negative self-priors remain unresolved. The author suggests the term “hyperactive inference” to explain compulsions as excessive Bayesian updating driven by inaccurate priors and a futile attempt to alleviate uncertainty about the self. The temporary relief of compulsive rituals ultimately reinforces the negative schemas generating pathological doubt and anxiety. Effective treatment should target core cognitive representations of the self rather than just surface symptoms.

In light of the findings of this Research Topic, OCD may benefit from several investigative approaches in order to gain further insights for assessment, diagnosis and treatment, ranging from clinical psychological conceptualization to neuropsychological and psychophysiological studies. These different methodological approaches conjointly may contribute to assist researchers and clinician to better face a challenging disorder such as OCD, and to provide relief for patients in functioning, well-being and quality of life.

## Author contributions

AP: Writing – original draft, Writing – review & editing. DC: Writing – review & editing. MG: Writing – review & editing.
